# Precision Feeding of Feedlot Calves Based on Phenotypic Production Profiles I. The Effect on Economic Important Production Parameters

**DOI:** 10.3390/ani15101361

**Published:** 2025-05-08

**Authors:** Andreas H. R. Hentzen, Dietmar E. Holm

**Affiliations:** Department of Production Animal Studies, Faculty of Veterinary Science, University of Pretoria, Private Bag X04, Onderstepoort, Pretoria 0110, South Africa; dietmar.holm@up.ac.za

**Keywords:** sustainable cattle production, cattle feedlot, precision cattle farming, feed efficiency, phenotypic production profiles

## Abstract

Precision feeding feeder calves according to their production potential could improve economically important production traits. We report two experiments, in two separate locations where feeder calves were categorized into previously established production profiles (PP 1, PP 2, and PP 3). These production-profiled calves were then randomly allocated to a high-producing diet (HPD), medium-producing diet (MPD), or a low-producing diet (LPD). The findings of experiment 1 (*n* = 430) were used to adjust the formulation of the diets in experiment 2 (*n* = 104). The mixed modelling of economically important feedlot carcass growth traits was performed in order to evaluate the interactions between PP classification and diet. PP 3 was repeatedly outperformed in all production parameters (*p* < 0.01) by PP 1 and PP 2, irrespective of the diet. In experiment 1, PP 1 calves outperformed PP 2 and PP 3 calves; however, the HPD was insufficiently matched to their growth potential. In experiment 2 where the metabolizable energy was increased, PP 1 calves gained 23.0 kg more carcass (*p* = 0.05), at an improved carcass feed conversion of 0.91 kg/kg (*p* = 0.06), when compared to PP 2 calves on MPD. This is the first report demonstrating the feasibility of precision livestock feeding based on the predicted growth needs of incoming feeder calves as determined by production profiling.

## Simple Summary

In most feedlots today, calves are not fed based on their individual potential to grow. This one-size-fits-all approach can miss opportunities to improve both animal performance and profits. Our study explores a smarter way to feed cattle—based on *precision livestock feeding*—where diets are matched to individual calves’ growth potential. We sorted incoming feeder calves according to their production potential (PP) using our methodology previously demonstrated: Group PP1 were calves with an above-average potential to grow, group PP2 were calves with an average growth potential, and group PP3 were calves with a below-average growth potential. Each group of calves was then fed a high-production diet, a medium-production diet (representing the current feedlot diet), and a low-production diet. Across all types of diets, calves in the PP1 group consistently performed the best (in terms of carcass gain), while PP3 calves lagged behind. However, we found that PP1 calves only reached their full potential when they were given a high-energy diet tailored to their needs. This research shows that precision feeding—giving each calf the right diet based on its growth potential—can work in real feedlot settings and is a promising step towards more efficient and profitable cattle farming. However, more work is needed in order to fine-tune the diets and feeding strategies for each group.

## 1. Introduction

As part of precision farming, managing livestock is one of the current challenges for agriculture [1]. Precision livestock farming (PLF), a term that appeared in the early 21st century with the first PLF conference held in 2003 [2], follows an innovative production system approach [3], which plays a key role in the fourth industrial revolution [4]. Precision livestock farming is potentially one of the most powerful developments and has the potential to revolutionize the livestock farming industry, together with artificial intelligence (AI), machine learning, and other technologies [5]. The three pillars of sustainability that PLF focuses on are social (human society), economic (production), and environmental (the monitoring of emissions and other impacts) [6,7]. This research focuses on the financial pillar of PLF in the feedlot, namely, precision feeding through a profit maximizing reformulation [8].

Precision livestock feeding, on the other hand, has multiple meanings. The efficient input approach to obtaining maximum and cost-effective outputs is recognized [7]. In this study, the focus is on feeder calves that have been categorized into established production profiles and we investigate three separate dietary interventions and their effect on the PP classification [9]. Precision animal nutrition, or precision feeding, is an integrated information-based approach to optimize the supply and demand of nutrients to animals in real time for target performance, profitability, product characteristics, and environmental outcomes [10,11]. This practice can significantly reduce protein intake by 25% and nitrogen excretion by 40% while increasing profitability by nearly 10% [11]. Precision feeding not only improves feed efficiency and animal welfare but also addresses environmental sustainability by reducing feed wastage and nutrient excretion, thereby mitigating greenhouse gas emissions and water pollution [12].

Precision feeding aims to match the nutrient supply precisely with the nutrient requirement of a feeder calf [13,14]. In the feedlot industry, more often than not, a feeder pen is made up of feeder calves that have different growth potentials. These animals are then fed with an average diet. Very little is published in terms of precision feeding in feeder calves. When growth maximization is the objective of the commercial production system, like the feedlot, nutrients must be provided at a level that will allow the most nutrient-demanding animals in the group to express their growth potential [15]. The current practice of providing a common diet to the group in the pen does not ensure a maximum economic return for all the animals in a sustainable manner. i.e., poor economic efficiency [15]. The potential can be unlocked by precision feeding to the potential, and this promises to affect the value of gain, reduce the cost of gain, improve the economic returns, and contribute to the economic pillar of sustainable livestock production.

The feed, after the purchase price of the feeder calf, is the second highest contributor to the total cost of producing a beef carcass [16,17,18]. Tailoring the supply, quality, and quantity of the nutrient supply to the requirements for maximum profit further supports the economic pillar of sustainable livestock farming.

The literature contains numerous publications on general agricultural precision farming. This is a well-researched and already-implemented practice in agriculture. The poultry sector has some documented attempts at precision farming as far back as 2003 [2], and others have followed in the pig and dairy industries [7,19]. With the application of precision feeding in the pig production system, an increase in profitability of nearly 10% has been reported [19], which emphasizes the inherent potential of such an approach. To our knowledge, the feedlot industry has no published information available. Venter, in her master’s thesis (2019), investigates the precision feeding of Nguni steers under feedlot conditions [20]. She uses different diets, different roughage inclusions, and different metabolizable energy levels to establish the best fit for the growth requirement of the Nguni breed in a South African feedlot. Subsequently, Linde et al. (2023) investigated the gene expression of South African beef breeds (Bonsmara and Nguni) fed different diets and found that genes are expressed differently under different dietary conditions for both breeds, with little breed interaction [21].

**Figure 1:**
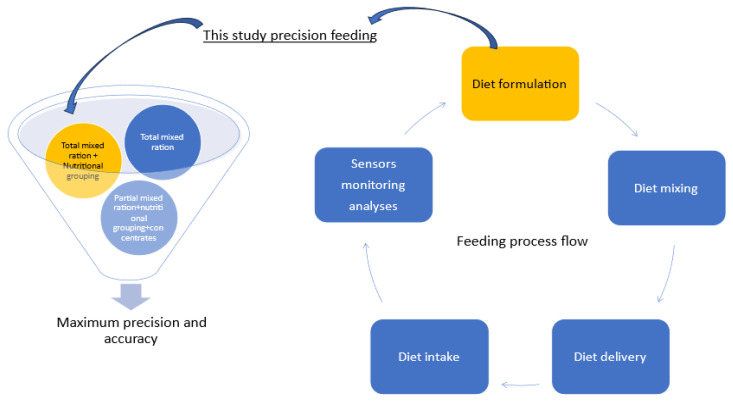
Schematic representation of the relevance of precision feeding in this study.

The nutrient supply is by diet formulation and macro composition only. Macro-composition is the matching and changing of raw materials in the diet formulation for growth with energy and protein as the main consideration [22,23] and as discussed in the Nutrient *Requirements of Beef Cattle Eighth* revised edition. This is carried out by formulating diets for maximum precision for growth [24,25,26], which has more recently been challenged by refocusing on maximum profit [8]. This does not include micro compositions like enzymes, microminerals, and other micro-ingredients that support growth and form part of a balanced diet for this study. In the process of achieving the maximum growth and accuracy of precision feeding for growth, this study is at the point of feeding a total mixed ration and nutritional grouping. The nutritional grouping is based on the genotype that is expressed in the phenotype and categorized in the PP [9]. This study does not consider precision feeding aspects related to the feeding process flow, like diet intake, diet delivery, and diet mixing. See Figure 1. This study addresses only one of the three pillars of sustainability, namely, the production (economic) pillar [7].

This study aims to establish the potential value of precision feeding production profiled feeder calves in the feedlot.

The objectives of the study are as follows:

- To establish the principle of precision livestock feeding, by matching the nutrient supply to growth potential;
- To prove the value of the precision livestock feeding concept, based on some economically important feedlot production traits;
- To establish the relevance of implementing precision livestock feeding in the South African feedlot industry.

The successful realization of the objectives will make a meaningful contribution to the South African feedlot industry and possibly globally.

## 2. Materials and Methods

This study is a prospective observational study in two South African beef feedlots and was approved by the Animal Ethics Committee of the University of Pretoria (AEC no 192-21 and 051-23).

The categorization of the feeder calves into production profiles before the feeding period was carried out per Hentzen and Holm [9]. In short, the animals were classified into production potential groups based on the composite observable characteristics or traits (phenotype) of muscular and skeletal development, ratios thereof, and body capacity. Muscular and skeletal development, which is better than average, was classified as PP1, whereas PP2 represented animals with average production potential. PP3 had the poorest muscular and skeletal development, which predicts below average feedlot production.

Feeder calves enrolled in this study were fed three different feedlot diets, formulated by feedlot nutritionists, registered with the South African Council for Natural Scientific Professions (SACNASP), and fed according to general feedlot norms. The average diet, medium-producing diet (MPD), was the diet formulated to general feedlot norms and considered the norm for average feeder calves. It was the diet fed in the respective feedlots and was considered as the control diet. The high-producing diet (HPD) was formulated to supply nutrients in a balanced feedlot diet, that supports additional growth. The low-producing diet (LPD) was formulated to supply nutrients for the demand of the slow-growth cattle.

To establish the value of PP in feeder calves, and data optimization, a two-experiment approach was considered. The goal of both experiments was the same: to generate data that support the value of PLF in beef feedlots and move away from feeding to the average animal. Experiment 1 was performed in a commercial feedlot. Results from experiment 1 were analyzed and considered in the reformulation of diets for experiment 2. In experiment 2, we fed animals by PP class and treatment (diet) in individual and grouped pens at an experimental farm. This approach had the additional advantage of analyzing on a per-pen basis. Furthermore, data regarding the economic value of feeding to the PP within a pen provided PP-specific intake data and feed costs, and, therefore, contributed to the economic analysis [27]. This was not possible in experiment 1. To our knowledge, experiment 2 is the first time animals were fed different diets, per PP classification. We could not find a suitable-sized feedlot that could accommodate both experiments in one location, and in one study, hence the two-experiment approach.

### 2.1. Experiment 1

**Figure 2:**
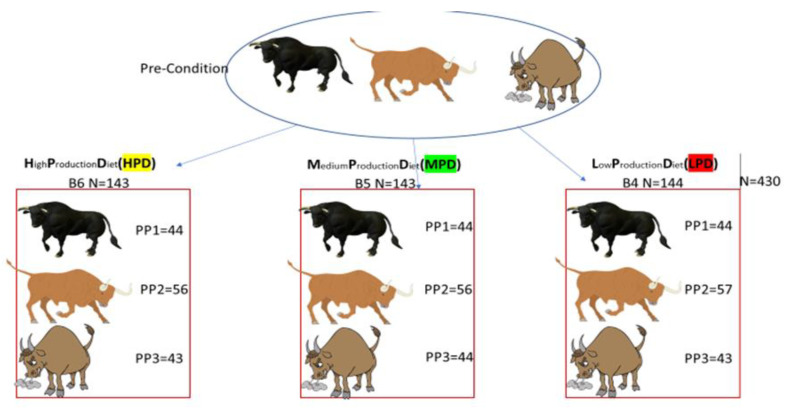
Diagrammatic representation of Precision Livestock Feeding Experiment 1.

In experiment 1, three feedlot pens (numbered B4, B5, and B6) in a commercial feedlot were each populated with an equal number of representatives of PP 1, PP 2, and PP 3 (Figure 2). PP 2 was made up of an equal number of PP 2+ and PP 2– animals. Each of the three feedlot pens was fed a different diet. Animals in pen B4 were fed the HPD, pen B5 the MPD, and pen B6 the LPD. This study design had the closest resemblance to a commercial feedlot.

#### 2.1.1. Phenotypic PP Classification

The study animals were individually evaluated by an experienced observer, who is a specialist veterinarian (the author), on their phenotypic appearances. The development of the PP classification system evolved over eight years. Other studies have demonstrated the repeatability of similar methodologies, which have been used in research [28,29]. The animal’s inclusion criteria for classification were the composite observable characteristics or traits (phenotype) of muscular and skeletal development, ratios thereof, and body capacity. Considering the phenotype presentation, each animal was categorized into one of the four production profiles (PPs) [9].

#### 2.1.2. Production Data Collection

On termination of the feeding period, all animals were harvested at a registered slaughter facility approximately 120–150 km from the feedlot. Outcome data points were collected, to analyze the data on carcass growth parameters on a per animal basis. Data collected for experiment 1 included the following [30]:

- Live gain in feedlot = Exit weight (kg)—entry weight (kg);
- ADG in feedlot = Gain (kg)/Days on Feed;
- Carcass entry weight = 0.694 × shrunk body weight—38.43 kg;
- Carcass gain (kg) = Carcass exit weight (kg) (measured)—carcass entry weight (kg) (calculated);
- Carcass ADG (CADG) = Carcass gain (kg)/Days on Feed.

#### 2.1.3. Animals

Our data represent feeder calves that were classified at the onset of the feeding period and followed prospectively to determine their growth performance during the feeding period. The study population represented the usual feeder calves from different origins that were procured by the commercial feedlot, and only male-gender calves were enrolled in the study. Before the onset of the study, the calves were prepared in a 60-day preconditioning phase at the same location [30]. The exact age of the animals was unknown and ranged from approximately seven to thirteen months, as is the industry standard in South Africa [16] for feeder calves, just weaned, to enter the feedlots. The animals were mixed beef breeds, as per the industry standard in South Africa [16].

Feeder calves (*n* = 430) had entry weights ranging from 261–313 kg (Table 4) and were first categorized into four PP categories, PP 1, PP 2+, PP 2–, and PP 3, which represent the production profile categories as described by Hentzen and Holm (2024) [9]. Subsequently, each PP group was randomly assigned to three feedlot production pens.

#### 2.1.4. Environment, Feeding, Housing, and Management of the Feeder Calves

The study was performed at a commercial cattle feedlot in South Africa, from spring to late summer. The facility is on degrees Latitude (DMS) 25°55′02″ S and Longitude (DMS) 28°35′52″ E, with an elevation of 1491 m above sea level with mean minimum and maximum spring and late summer temperatures of 12 °C and 29 °C, and 17 °C and 30 °C, respectively, in this area.

At the time of entering the preconditioning period and subsequent placement into the feedlot, all animals were vaccinated and treated for internal and external parasites, as per the feedlot prescribed protocol (Table 1). The implant strategy was followed, as per the prescribed protocol (Table 1). This is the exact protocol Hentzen and Holm (2024) followed in their proof-of-concept study [9].

After PP categorization, animals were individually weighed before block-randomizing them by PP category and weight into the three diet groups. The PP 1 category consisted of *n* = 132 and was randomly placed into the HPD (*n* = 44) Pen B4, MPD (*n* = 44) Pen B5, and LPD *(n* = 44) Pen B6 diet groups. Similarly, PP 2+ animals were randomly assigned to HPD (*n* = 28), MPD (*n* = 27), and LPD *(n* = 28), and PP 2– animals were randomized to HPD (*n* = 29), MPD (*n* = 29), and LPD (*n* = 28). The PP 3 animals (*n* = 129) made up the rest of the experimental animals and were randomized into the three treatment groups, respectively, *n* = 43 each. PP 2+ and PP 2– were classified into separate production profile groups to equally contribute to the PP 2 (representing the average) group (Figure 2).

A balanced starter diet was formulated and fed for the first 14 days to all experimental feeder calves (B4–B6) before commencing with the experimental diets. The starter diet was formulated to achieve a protein content of 14–16% crude protein (CP) and metabolizable energy (ME) of 10.5 MJ/kg DM. After the general starter diet, the three different diets were introduced (LPD in B4, MPD in B5, and HPD in B6).

The cattle were housed and kept in three purposefully designed rectangular feedlot pens (30 m × 60 m) with a feeding trough on one side. Animals were fed in groups not exceeding 145 animals per pen and with a population density ≥12 m^2^/animal. The pens were adjacent to each other, in the middle of a commercial feedlot production line; in other words, none of the experimental pens were at the end of a line. This was carried out to minimize the impact of any pen effect, seeing that pen and diet were collinear in this experimental study design. Feedlot pens at the extremities of a production line are known to cause more variance in cattle performance, compared to pens in the middle of a line [31]. Furthermore, the pen where MPD was fed (B5) was situated between the pens where LPD and HPD were fed (B4 and B6, respectively), so that both experimental pens (B4 and B6) had pens on either side where the average diet for cattle in this feedlot was fed (MPD).

Animals were fed twice daily according to the intake of the previous days to a zero-bunk score [32], and had continuous access to clean, fresh water. The pens were cleaned of excess cattle dung once every month or when necessary. Animals in each pen were observed twice daily for signs of disease and discomfort. Animals identified as sick or in discomfort were removed from the pen and the study, and treated according to the overseeing veterinarian’s protocol. Animal welfare and management, including health management, was according to prescribed protocol and/or best management practices.

Due to logistical reasons, the slaughter of the animals occurred over 12 separate days. On the day of slaughter, the same number of animals per PP classification was randomly slaughtered from each pen. In other words, cattle were not individually selected based on market readiness but were fed to the common market endpoint, grade A2/A3 [33] as a group.

#### 2.1.5. Diets

The three diets were formulated using cattle feed formulation software based on the Cornell Net Carbohydrate and Protein System (CNCPS version 6.5). The already existing diet in the feedlot was used for the MPD and is considered a typical diet for the South African feedlot industry (feeding to the pen average). This diet was fed to pen B5. The HPD and LPD used in experiment 1 were formulated using the MPD as a base. The HPD, fed to pen B6, was formulated in an attempt to address the higher production potential of incoming feeder calves with PP 1 classification. Based on González review, the HPD, therefore, included additional energy and made use of rumen-protected proteins (amino acids) to support additional growth potential [34]. The LPD was lower in ME, higher in peNDF, and lower in rumen-degradable protein (Table 3). Pen B4 received the LPD, formulated to match the nutritional needs of feeder calves with poorer growth potential (represented by PP 3). The chemical composition of the three diets is summarized in Table 2.

This approach resulted in having three pens (B4; B5; and B6) next to each other, in the middle of a feeding line. This was carried out to avoid experimental pens being on the end of a feeding line and, thus, being exposed to different environments. The three trial pens were flanked by other feedlot pens, B3 and B7.

The feed is the intervention, and the reaction of the different PP classification animals to the diets is the data captured to address the hypothesis. (Table 2).

### 2.2. Experiment 2

In experiment 2, similar PP animals were fed together in smaller feeding pens, keeping to a density of ≥12 m^2^/animal. Here, the same PP classification animals were fed together and exposed to the HPD, MPD, and LPD. The MPD, as in experiment 1, was a ration fed to a commercial feedlot and considered a control diet. This approach made it possible, in experiment 2, to add an analysis per pen, representing the same PP classification, which was not possible in experiment 1. By doing so, feed intake per PP classification and per diet could be determined, allowing for economic implications to be analyzed.

#### 2.2.1. Phenotypic PP Classification

The study animals were individually evaluated by an experienced observer, who is a specialist veterinarian (the author), on their phenotypic appearances. The development of the PP classification system evolved over eight years. Other studies have demonstrated the repeatability of similar methodologies, which have been used in research [28,29]. The animal’s inclusion criteria for classification were the composite observable characteristics or traits (phenotype) of muscular and skeletal development, ratios thereof, and body capacity. Considering the phenotype presentation, each animal was categorized into one of the four production profiles (PPs) [9].

#### 2.2.2. Production Data Collection

On termination of the feeding period, all animals were slaughtered at a registered slaughter facility approximately 100 km from the feedlot. Outcome data points were collected, to analyze the data and establish the actual growth compared to the allocated PP classification at the beginning of the feeding period. Data collected were similar to that described for experiment 1, with the addition of the following data:

- Daily feed intake measured in kg on an as-is basis;
- Carcass feed conversion ratio (CFCR) = total kg of feed consumed (as is) (kg)/carcass gain (kg);
- The carcass feed cost of gain (CFCOG) is calculated as the monetary value of feed consumed to gain 1 kg of carcass = Tot R-value consumed/Carcass gain (kg) [8,31].

#### 2.2.3. Animals

In experiment 2, animals were sourced in the same way as described for experiment 1, except that the animals were sourced from and the 60-day preconditioning phase was completed at a different commercial feedlot, approximately 350 km from the experimental farm where the study was performed, and feeder calves (*n* = 108) had entry weights ranging between 161–260 kg.

#### 2.2.4. Environment, Feeding, Housing, and Management of the Feeder Calves

The study was performed at a registered research facility in South Africa from mid-summer to autumn. The facility is on the degrees Latitude (DMS) 28°13′34” S and Longitude (DMS) 28°30′09” E, with an elevation of 1813 m above sea level with mean minimum and maximum mid-summer and autumn temperatures of 15 °C and 25 °C, and 12 °C and 25 °C, respectively, in this area.

Preconditioning, vaccination, and implant protocols were similar to experiment 1 (Table 1).

Feeder calves in experiment 2 were individually identified, weighed, and categorized into the four production profiles (PP 1; PP 2+; PP 2–; and PP 3) [26] at the original commercial feedlot. Each of the four PP classifications consisted of 27 animals that were randomly allocated on arrival at the experimental farm, after ranking based on weight, into 36 pens in total. Each PP category was represented in six pens with four occupants per pen, and three pens for individual animals. Within each PP category, the allocated pens were then subsequently randomized into three diets, using a random number generated in Microsoft Excel (MS Office): HPD, MPD, and LPD.

**Figure 3:**
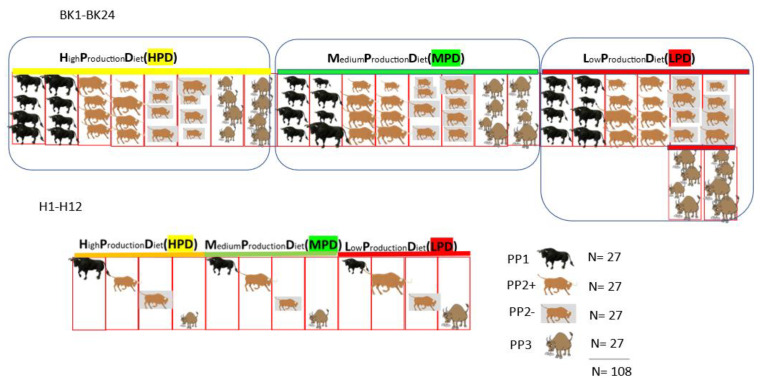
Diagrammatic representation of animal allocation to the different pens in Precision Livestock Feeding Experiment 2.

This approach resulted in having two grouped pens of each PP classification (1; 2+; 2–; and 3) on the LPD, MPD, and HPD (BK1 to BK 24 in Figure 3). The individual holding pens were similarly allocated, with each pen holding only one animal (H1 to H12 in Figure 3). Experiment 2 was carried out to address and support possible data shortfalls of experiment 1 and allowed for the reformulation of the diets (Figure 3).

The cattle were housed and kept in 24 purposefully designed rectangular feedlot pens (4 m × 9 m) with a feeding trough on one side. The individual holding pens are of a similar design, but smaller (2 m × 4 m). Animals were fed twice daily according to the intake of the previous days to a zero-bunk score [32] and had continuous access to clean, fresh water.

The pens were cleaned of excess cattle dung once every month or when necessary. Animals in each pen were observed twice daily for any signs of disease and/or discomfort. Animals identified as sick or in discomfort were removed and treated according to the overseeing veterinarian’s protocol. Animal welfare and management, including health management, was according to prescribed protocol and or best management practices.

All cattle were slaughtered on the same day and at the same slaughter facility, as is commonly the practice in large commercial feedlots in South Africa. In other words, cattle were not individually selected based on market readiness but were fed to the common market endpoint, grade A2/A3 [33] as a group.

#### 2.2.5. Diets

The starter diet was formulated similarly to that of experiment 1. After the general starter diet, all animals were individually weighed, placed back into their respective pens, and started subsequently on the three different diets, LPD, MPD, and HPD. Based on our results from experiment 1, the energy in the HPD of experiment 2 was increased further. The ME in the LPD was further reduced, to widen the gap between MPD and LPD from that in experiment 1. Put differently, rations were reformulated to accentuate the interaction between diet and PP classification on feedlot performance.

The three experimental diets were formulated with the CNCPS beef model considering relevant environmental, managerial, animal, and nutritional factors. A 200 g difference in live ADG between the three diets was targeted mainly by using a rumen-protected fat supplement (Megalac^®^, Nutribase, Pretoria, South Africa) to create differences in metabolizable energy gain. Metabolizable protein was adjusted to ensure that metabolizable protein gain was not first limiting. LPD and HPD were formulated for feeder calves with poorer and better growth potential compared to the industry norm. The three diets’ chemical composition is summarized in Table 3.

### 2.3. Statistical Analysis

Data for both experiments were captured in a spreadsheet and transferred to STATA 14.0 (Statacorp, College Station, TX, USA) for analysis. The analysis of variance between means of the series of measurements collected and the digital model was carried out by one-way ANOVA, using the Bonferroni test to estimate the significance of differences between means. Results were considered statistically significant if *p* < 0.05.

In experiment 1, subsequent mixed-effects regression models for carcass and growth outcomes were constructed, adjusting for possible confounding factors, with the calf as the experimental unit, diet as fixed effect, and pen as a random effect. Initially, all possible covariates were included in the model, and removed one by one from the model based on the highest Wald *p*-values. When only independent covariates (with Wald *p*-values < 0.05) remained in the models, potential confounding of other covariates was tested and considered significant if the addition of a potential confounder changed the coefficient of any independent covariate by ≥15%. The exception was carcass entry weight, which was forced into all models due to the known associations between entry weight and feedlot production [35]. An interaction term between diet and PP classification was included in the model and kept if significant interaction existed. Validity of the models was confirmed by the chi-square statistic, to estimate the overall effect of the model on the dependent variable and was considered a good fit if *p* < 0.05. For the mixed-effects models, PP 2+ and PP 2– were combined as PP 2, representing the average feeder calf, to compare the carcass and growth outcomes of the better (PP 1) and weaker (PP 3) calf to that of the average (PP 2) calf after adjusting for possible confounders, on the different diets (LPD, MPD, HPD).

In experiment 2, Days on Feed (DoF) was not considered a covariate because it was similar for all animals. In experiment 2, feed intake and carcass feed conversion were determined for each PP classification and diet, using the pen as an experimental unit rather than the animal, because feed intake could only be measured per pen, and not per animal, in the case of the grouped pens. Following this, a similar mixed-effects model to those described in experiment 1 was created for carcass feed conversion, which also considered the number of animals in a pen as an additional potential confounder.

## 3. Results

### 3.1. Experiment 1

#### 3.1.1. Population

No deaths were recorded. Only one animal was treated for bovine respiratory disease (BRD) from pen B5 and removed from the study. Three lame animals were removed from the study for nonspecific infectious lameness, one from Pen B4 and two from Pen B6.

#### 3.1.2. Feedlot Production Data

Male feeder calves that made it to slaughter and of which data were eligible for analyses were 386. The data that were not considered for analyses included data of animals which had a carcass growth rate (CADG) of more than 2 standard deviations of the mean, 1.28 kg/d. The data deviations were considered to be a result of recording errors and were eliminated (*n* = 8 in pen B4; 4 in B5; and 2 in B6). ID problems with the reading of the electronic tag at the slaughter facility made up the rest of the omissions (*n* = 26).

The mean entry weight differed significantly between PP classifications, with PP 1 being 51.75 kg heavier than PP 3 at entry (*p* < 0.01, Table 4). On the other hand, the mean entry weight per diet was not different (*p* = 0.89, Table 4). The carcass entry weights were calculated [30] and showed similar trends (PP classification effect *p* < 0.01 and diet effect *p* = 0.89).

The mean (exit) carcass weight (kg) differed significantly (*p* < 0.01) between the PP categories, and not between the diets (*p* = 0.25) (Table 4). Compared to the average feeder calf (PP 2), PP 3 had a mean carcass weight of 305.83 kg, being 37.09 kg less than that of PP 2. Production Profile 1, in comparison, had a carcass exit weight of 22.05 kg heavier than PP 2. The difference between the lightest carcass of PP 3 and the heaviest carcass represented by the PP 1 class was 59.11 kg (*p* < 0.01).

The animals were fed to the common SA market endpoint of A2/A3 classification based on fat deposition [29]. Following the feeding period, there were no animals with a fat grade below the desired A2 grade; however, there were four carcasses from animals in the HPD, five from the MPD, and six from the LPD graded as too fat (A4 grade).

All animals could not be slaughtered on the same day due to logistical limitations, resulting in the Days on Feed (DOF) being positively and significantly (*p* < 0.001) associated with carcass gain (kg). Days on Feed was, therefore, accounted for in all further mixed models.

After measuring the mean carcass weight gains (kg) in the feedlot, we found that the trend of PP 3 being the poorest-performing PP category continued. This time, both the effect of PP (*p* < 0.01) classification and diet (*p* = 0.01) were significantly associated with carcass weight gain (Table 4). Production Profile 3 calves gained the least carcass weight, 161.70 kg, and, using PP 2 as the baseline, PP 1 gained 9.96 kg more carcass weight (*p* < 0.01), and PP 3 gained 13.26 kg less carcass weight (*p* < 0.01) across all diets. The effect of diet on carcass weight gain was also significant: Animals on the HPD and MPD gained 8.75 kg and 8.40 kg more than those on the LPD over the feeding period, respectively (*p* < 0.05).

The rate of gain (ADG) was calculated on a live as well as carcass basis. Like mean carcass weight gains (kg), PP classification and diet both had significant effects on carcass average daily gain (CADG) (*p* < 0.01, Table 4). PP 1 and PP 3 calves had a CADG of 0.06 kg/d more (*p* < 0.05) and 0.10 kg/d less (*p* < 0.01), respectively, when compared to the average (PP 2). As with carcass weight gain (kg), the HPD and MPD had a better CADG, 0.07 kg/d (*p* < 0.01) and 0.06 kg/d (*p* = 0.02), respectively, when compared to the LPD.

In the mixed-effects models, the PP 2 feeder calves on the MPD were used as the reference group since they represent animals with an average production potential fed on an average diet in a commercial feedlot environment. The effect of carcass entry weight was significant (regression coefficient = 1.04; i.e., for every 1 kg heavier carcass entry weight, calves produced 1.04 kg more carcass weight at the end of the feeding period). Days on Feed also had a significant effect (regression coefficient = 1.73; i.e., for every day longer fed, carcass weight (kg) increased by 1.73 kg). After adjusting for the significant effects of the carcass entry weight and DoF, however, the multiple-regression model demonstrated that the effect of PP classification on the carcass exit weight remained significant even after adjusting for the effects of the carcass entry weight and DoF. Put differently, the carcass entry weight, DoF, and PP classification all had independent effects on the carcass exit weight.

Based on the mixed-effect models of carcass gain (kg) with the interaction between PP and diet, and PP 2 fed the MPD as the reference value, we observe that PP 1 did not gain significantly more carcass weight when fed on the HPD (*p* = 0.39). The PP 3 animals had a significantly lower carcass weight gain (kg) on all the diets when compared to the PP 2 fed the MPD (*p* < 0.01, Table 5).

When looking at the CADG with a similar model, it demonstrates that, when compared to the average PP 2 classification on the MPD, the PP 3 classification had a 0.09 kg/d lower CADG even when fed the HPD (*p* = 0.02). The PP 1 classification feeder calf never had a significantly higher CADG fed the HPD, MPD, or LPD when compared to the PP 2 fed the MPD. For the PP 2 fed the HPD, we see that the PP 2 CADG was only 0.16 kg/d better than the same class of animal fed an MPD (*p* = 0.65).

### 3.2. Experiment 2

#### 3.2.1. Population

During the feeding period, two animals were removed from the study because of re-accruing bloat, both PP 2 animals, one on the MPD in pen BK12, and the other on LPD in pen BK19. Both these animals’ data were omitted from the data set. Two animals died, one with Anaplasmosis (PP 1 on LPD, pen BK18) and the other because of bloat (PP 3 on MPD, pen BK13). No animals were treated for BRD.

#### 3.2.2. Feedlot Production Data

Male feeder calves’ data from 104 animals were analyzed. The animals were all slaughtered on the same day, after 142 Days on Feed (DoF), all having carcass grades of A2 or A3 [33].

The mean entry weight, exit weight, live weight gain (kg), ADG (kg/g), carcass entry weight, carcass weight, carcass gain (kg), and carcass rate of gain (CADG) were recorded (Table 6). The results presented in Table 6 represent measurements on individual animals as experimental units.

The mean lives and carcass entry weights followed a similar trend to that observed in experiment 1. The entry weight differed significantly on the PP classification (*p* < 0.01) compared to the diet effect, where no difference was seen (*p* = 0.80). Similar to experiment 1 and previous research by Hentzen and Holm (2024) [9], the entry carcass weight also differed significantly (*p* < 0.01) on the PP classification, and, like the mean entry live weight, there was no difference (*p* = 0.80) on the diet (Table 6).

The mean carcass weight was significantly affected by both the PP classification (*p* < 0.01) and the diet (*p* < 0.01). The PP 3 animals had a 55.15 kg lighter carcass (*p* < 0.01) than the PP 1. The average carcass weight of the HPD was 290.60 kg, which was 28.4 kg heavier (*p* < 0.01) than animals on the LPD (Table 6).

Measuring the mean carcass weight gains (kg) in the feedlot and comparing it to the feedlot average equivalent, PP 2, PP 3 gained 27.16 kg less (*p* < 0.01) carcass weight over the same period.

The mean CADG was significantly affected by both PP classification (*p* < 0.01) and diets (0.01, Table 6). The current trend of the study continued, with the PP 3 CADG being 0.19 kg/d lower (*p* < 0.01) and PP 1’s being similar to that of PP 2’s (*p* = 0.17, Table 6).

The captured feed intake and the carcass gain (kg) per pen enabled us to calculate the mean carcass feed conversion ratio (CFCR) per PP classification (PP 1; PP 2; and PP 3) and diet (LPD; MPD; and HPD). The intake of PP 2 classification was 9.31 kg/d on a dry matter basis. The average intake differed for different PP classifications (*p* < 0.01). There was no effect observed between the diets (*p* = 0.67). The mean CFCR differed between PP classification (*p* = 0.02) and diet fed (*p* < 0.01) when comparing pens (Table 7).

When looking at the mixed-effects model of carcass rate of gain (CADG) interaction between PP and diet, it demonstrates that, when compared to the PP 2 classification fed the MPD, the PP 3 classification had 0.15 kg/d less carcass rate of gain (CADG) even on the HPD (*p* < 0.01) and 0.17 kg/d less CADG on the MPD (*p* < 0.01), and 0.31 kg/d lower CADG on the LPD (*p* < 0.01). Different from experiment 1, the PP 1 classification feeder calf had, this time around, a significantly higher CADG of 0.16 kg/d (*p* < 0.01) on the HPD, when compared to PP 2 fed the MPD. When feeding the PP 1 animals the LPD, the CADG did not differ from that of PP 2 on MPD (*p* = 0.25).

The carcass gain (kg) was looked at with a similar model (Table 8). It is noteworthy to report that the difference between PP 1 fed the HPD and PP 3 fed the LPD is 67.27 kg in carcass gain (kg), i.e., sellable meat, over the feeding period using PP 2 fed the MPD as reference.

A similar model was used to investigate the carcass feed conversion compared to the feedlot average PP 2 fed the MPD (Table 9). It demonstrated that PP 3 on the LPD had a poorer feed conversion of 1.5 kg/kg (*p* < 0.01). Put differently, PP 3 fed the LPD needed 1.5 kg more feed to gain 1 kg of carcass. PP 1 fed the LPD also tended to need 0.90 kg more feed to gain 1 kg of carcass weight (*p* = 0.07). The trend of PP 3 being the poorest continued with the CFCR of PP 3 deteriorating as the diets ME decreased. When compared to PP 2 on the MPD, the CFCR of PP 3 fed the HPD, MPD, and LPD increased from 0.31 kg/kg, 0.68 kg/kg, and 1.5 kg/kg, respectively, meaning they had to increasingly consume more feed, as the ME decreased to gain the same kg of meat.

## 4. Discussion

This study aims to demonstrate the value of PLF based on the growth potential of feeder calves, as visually classified according to Production Profiles (PPs) before entering the feedlot phase. This approach supports and contributes to the economic pillar of sustainable PLF. The environmental and social aspects of PLF are relevant in the feedlot industry but are not included in this study. The potential impact of PLF in alleviating methane reduction, and the reduction in nitrogen and phosphate in the environment are valid and recognized, but also not included in this study. The potential role that PLF can have on market segmentation is also valid, but not part of this research.

This study focuses on the matching of the nutrient supply to the nutrient requirements of feeder calves fed a balanced diet. It is acknowledged that diets need refinement to unlock the full potential of PLF. The authors do not advocate the specific diets fed in this study to be the only ration for precision feeding, but rather the principle of matching the nutrient supply to growth potential. Our focus was on energy (ME) and protein, since they are the main contributors to growth [27,36].

The need to classify feeder calves according to production potential, and then feed accordingly, has been expressed [9]. Successfully categorizing feeder calves into Production Profiles [9] before the feeding period is the initial step in PLF. The feeder calves with better Production Profiles can potentially add additional value to the feedlot when an additional supply of nutrients is attained, for the demand of additional growth [27]. Similarly, losses incurred by calves with below average production potential could potentially be mitigated by PLF.

### 4.1. Demonstrating the Principle of Precision Livestock Feeding, by Matching Nutrient Supply to Growth Potential

The MPD in both experiments are diets that are fed in commercial feedlots and are used as the reference. They are good balanced diets, established through positive feedback over the years. Since carcass gain is the product of CADG and DoF, the discussion focuses on the carcass gained by different PPs on different diets. Compared to the average animal (PP 2 fed on an average diet MPD), the PP 2-classified feeder calves did not gain additional carcass weight over the feeding period when fed the HPD in experiment 1 and 2 (1.9 kg and 5.5 kg difference, *p* = 0.696 and *p* = 0.437, respectively), accounting for confounders. In both experiments, the higher ME diets failed to improve the carcass gain significantly, measured over the feeding period, and came at a higher cost. When the PP 2 were fed the LPD, they gained 10.7 kg less carcass weight (*p* < 0.03), compared to when they were fed the MPD in experiment 1. The same class of animal, PP 2, in experiment 2 gained 16.78 kg less carcass than the PP 2 on the control diet (*p* = 0.01). It is, therefore, evident that, in both experiments, the nutrient supply of the MPD is a good fit for the PP 2 growth potential, confirming our initial suspicion that the MPD base diet was established over many years to suit the production potential of the average feeder calf.

The PP 3 classification animals are animals with a poor growth potential, as seen in the results of the two experiments and previous research [9]. Feeding this class of animals with the HPD, compared to the average (PP 2 fed an MPD), shows a growth response to the nutritional supply in both experiments 1 and 2. In experiment 1, the carcass gain of PP 3 animals was 19.17 kg, 14.93 kg, and 12.07 kg less than that of the controls when fed the LPD, MPD, and HPD, respectively (*p* < 0.03, Table 5). A similar trend was observed in experiment 2 where PP 3, when fed the LPD, gained 44.27 kg less carcass weight; when fed the MPD, gained 23.37 kg less carcass; and, when fed on the HPD, gained 21.37 kg less carcass weight compared to PP 2 on the MPD (*p* < 0.02, Table 8). The PP 3 feeder calves do, therefore, respond to the additional nutrient supply but at an additional cost and remaining below the performance of PP 2 calves on the MPD. Based on both experiments’ data and the respective diets fed, it is, therefore, not recommended to feed PP 3 the LPD, although further investigation is needed to determine the financial implications for a feedlot.

The PP 1 classification animal, having the highest growth potential [9], did not show a positive response to the higher nutrient supply in experiment 1: The PP 1-classified calves on the MPD had an additional carcass weight gain of 9.84 kg (*p* = 0.06); however, the carcass weight gain of PP 1 calves on the HPD and LPD did not differ from that of PP 2 calves on the MPD (+4.43 kg and −3.14 kg, and *p* = 0.39 and *p* = 0.54, respectively). On the contrary, in experiment 2, the reaction of the PP 1 class on the HPD of +22.00 kg (*p* < 0.01) is noteworthy where the ME was significantly increased by the addition of rumen-protected fat (Table 3). In this experiment, the PP 1 calves fed the MPD or LPD did not gain significantly more or less weight than PP 2 calves on the MPD (+1.8 kg and −9.66 kg, *p* = 0.83 and *p* = 0.25, respectively, Table 8). This is in line with the results [20] found in her precision feeding research in South Africa, where a ration higher in ME resulted in a significant improvement in ADG live, gain kg live, and carcass weight [15]. The additional 22 kg carcass weight gain for PP 1 calves on the HPD comes at a higher diet cost; however, compared to experiment 1, it is a better match to the diet. Financial implications need to be considered in the future.

The PP 1 growth potential is not a good match to the LPD in both experiments, and, based on these two experiments, PP 1 should not be fed an LPD.

Overall, we found, in experiment 1, that the effect of PP classification on carcass gain was more significant than the effect of the three different diets (Table 5). This is why the diet formulation was then reformulated for experiment 2, where the improved matching of the HPD with the PP 1-classified calves, in particular, demonstrates the principle of precision livestock feeding, by matching the nutrient supply to growth potential.

### 4.2. The Value of Precision Livestock Feeding, Based on Some Economically Important Feedlot Production Traits

The value of supplying the nutrients that support the growth potential lies in the reaction of feeder calves classified according to PP, as measured by economically important growth traits [27]. The rate of carcass gain (kg) and the efficiency of the gain (kg feed: kg gain ratio) are the most important factors to consider and are discussed. The more carcass weight gained, the more saleable kg meat, resulting in a higher revenue. Better efficiency means that the cost of carcass gain is reduced, thereby also providing a higher revenue.

The feeder calves with the highest PP classification add the highest value when fed the highest production diet. This is seen in experiment 2 where PP 1 calves fed the HPD had a CADG of 0.16 kg/d higher than PP 2 on the MPD (*p* < 0.01). This value was unlocked after the reformulation, based on the results of experiment 1. The value lies in the possibility to estimate the production potential at the start of the feeding period by PP classification, and subsequent feeding to that potential of growth.

The trend is similar when comparing the CADG, carcass gain (kg), and resulting carcass weight (kg) on the ANOVA analyses. The PP classification has a significant effect on the production parameters in both experiments (*p* < 0.01). Comparing the diet effect in the two experiments, the effect of the higher ME in experiment 2 resulted in a significant (*p* < 0.01) improvement in all production parameters (Table 6). The growth rate was always lower when feeding PP 3 compared to PP 1 and PP 2, independent of what diet they were fed (*p* < 0.02). Different to PP 1, it is difficult to unlock the value of a PP 3 animal through the diet, based on this research, and this supports that a poor animal cannot be fed right (Table 7 and Table 9). To feed the PP 3 profitably, factors other than production and diet need to be considered. This could include an improved diet formulation to ensure a better cost of gain or address the anticipated production loss during procurement.

Another factor that could not be considered in this research that may have influenced the results is the age of the incoming feeder calf. In the current South African feedlot industry, this information is not readily available. Future studies should investigate whether age may influence an incoming feeder calf’s response to different diets, within PP classification. When the information is available, age may therefore further improve our ability to match the diet to the nutrient requirement of the incoming feeder calf.

The efficiency measure, CFCR, could only be measured in experiment 2. The rate (*p* < 0.01), as well as the efficiency (*p* = 0.06) of gain, is superior in the PP 1 animal fed the HPD (which has been designed in an attempt to support its superior production potential). This makes PP 1 calves ideal candidates to further reformulate diets and challenge the feed. Considering the substantive influence feed conversion has on economic returns [27], the LPD might not be a diet to consider in circumstances comparable to experiment 2.

From our data in experiment 2, where the diets were more extreme in terms of energy and protein content, it is evident that the efficiency of growth is more severely impacted by the LPD in calves with an average or low production potential than in calves with a better production potential. The PP 2 and PP 3’s CFCR on the LPD was 1.25 kg/kg (*p* < 0.01) and 1.50 kg/kg (*p* < 0.01) lower than that of PP 2 on the MPD after adjusting for entry weight, respectively (Table 9). This means that the PP 2 and PP 3 calves consumed 1.25 and 1.50 kg more feed for every 1 kg carcass weight gained when fed the LPD, compared to PP 2 calves fed the MPD. On the other hand, calves with the PP 1 classification seemed to tolerate the lower energy and protein in the LPD better, with a smaller (and not significant) difference in CFCR when compared to PP 2 on the MPD (0.87, *p* = 0.07) (Table 9). On the other end of the spectrum, the HPD did not seem to significantly improve the efficiency of growth in any of the PP classifications, although it is evident that CFCR is most likely to improve more for PP 1 calves on the HPD (0.91 kg/kg better CFCR for PP1 on the HPD compared to PP2 on the MPD, *p* = 0.06) when compared to PP 2 and PP3 calves where there was no improvement in feed efficiency evident at all when fed the HPD (*p* = 0.26, and *p* = 0.50, respectively). This, once again, implies that a diet with a higher energy and protein content may only be able to unlock additional growth efficiency from animals where such an ability is inherent.

### 4.3. The Relevance of Implementing Precision Livestock Feeding in the Feedlot Industry

The beef feedlot industry must base the decision-making on carcass-based measurements [37], and act on the influence the diet has on the growth rate [38]. This study has generated additional information, in that PP-classified animals respond to precision livestock feeding, which aligns with Tatum’s findings. The improved growth rate and reaction to the additional nutrient supply is relevant; practical implementation in a cost-sensitive industry calls for some financial indicators.

The absence of precision feeding in cattle feedlots is a constraint to sustainable beef production. The data of these two experiments show that identifying the individual with the highest CADG and lowest CFCR potential before the feeding period is essential and supplying these individuals with the nutrients that support that specific CADG and CFCR is possible. Matching the cost of those nutrients to the relative growth potential in a cost-effective way makes feeding to the production potential in the intensive feed industry relevant.

Further research is needed in order to investigate the financial implications of precision feeding based on PP classification, which depend not only on the growth rate and efficiency of growth but, amongst others, also on the cost of the diet and the cost of standing for a longer time in the feedlot. Countries with similar feeder calves and variations in receiving feeder calves will find value in this research and support the further research in and application of precision livestock feeding. The second highest cost component of a beef feedlot (feed cost) can be reduced by improving the feed efficiency, which improves the greater economic returns, like the margin over feed cost. The concept of PLF promises greater efficiency in resource utilization [39]. This leads to possible reduced excretions of, amongst others, nitrogen (N) and methane [7], and a possible reduction in the carbon footprint [40].

## 5. Conclusions

This is the first report to demonstrate the principle of precision livestock feeding in a commercial feedlot setting based on the Production Profile (PP) of feeder calves following phenotypic appraisal at feedlot entry. It is concluded that precision livestock feeding, by the matching nutrient supply (ME and protein) with feedlot growth potential, has value in the South African feedlot environment, based on the improved carcass growth and efficiency of growth.

This study is fundamental in contributing to future studies that build on this groundwork in precision livestock feeding in feedlots. This study also shows the value and the relevance of feeding to the potential of production groups. This new information is applicable in commercial feedlots and supports the sustainability of the intensive feeding industry.

## Figures and Tables

**Table 1:** Summary of processing protocols at the start of preconditioning and the feeding phase.

	Preconditioning	Processing at the Feedlot
Viral vaccination	MLV ^1^ 5-way	MLV ^1^ 5-way
Respiratory bacterial vaccination	*Mannheimia haemolytica* (leucotoxin)	*Mannheimia haemolytica* (leucotoxin)
Other bacterial vaccinations	7-way clostridial (toxoid), botulism (toxoid) and anthrax (avirulant live)	10-way (toxoid)
Endo- and ectoparaciticides	Endectocide (1% ivermectin)	Endectocide (1% doramectin), topical pyrethroid pour-on
Metaphylaxe	-	Depending or perceived risk per pen, either oxytetracycline or tulathromycin, or none
Growth promoter implant	40:8 TBA ^2^: Oestradiol	200:28 TBA ^2^: Oestradiol

^1^ Modified live virus containing modified live strains of Infectious Bovine Rhinotracheitis (IBR), Bovine Viral Diarrhea (BVD type 1 and 2), Para Influenza Virus (PI3), and Bovine Syncytial Virus (BRSV). ^2^ Trenbolone Acetate.

**Table 2:** Experiment 1: Chemical composition of experimental diets on a 100% DM basis.

	LPD^4^		MPD^5^		HPD^6^	
	Grower	ZPHCL^7^	Grower	ZPHCL^7^	Grower	ZPHCL^7^
Crude Protein (%)	11.8	11.9	13.9	13.0	13.0	11.9
NDF^1^ (%)	13.2	12.4	12.8	12.1	12.3	11.8
RUP^2^ (%)	36.1	41.9	43.4	41.9	45.6	44.1
ME^3^ (MJ/kg)	11.1	11.1	10.8	11.0	10.9	11.1

NDF^1^ Neutral Detergent Fibre. RUP^2^ Rumen-Undegraded Protein. ME^3^ Metabolizable Energy. LPD^4^ Low-Production Diet. MPD^5^ Medium-Production Diet. HPD^6^ High-Production Diet. ZPHCL^7^ is Zilpaterolhydrochloride from Virbac SA (Grofactor^®^).

**Table 3:** Experiment 2: Chemical composition of experimental diets on a 100% DM basis.

	LPD^1^ *	MPD^2^ *	HPD^3^ *
Crude Protein (%)	13.0	14.0	15.0
Neutral Detergent Fibre (%)	26.6	22.2	21.2
Starch (%)	42.6	45.3	41.3
Fat (%)	2.6	3.2	5.8
Metabolizable Energy (MJ/kg)	10.6	11.3	12.0
Ration Cost (R/kg)	5.01	5.37	6.37

* ZPHCL is Zilpaterolhydrochloride from Virbac SA (Grofactor^®^). LPD^1^ Low-Production Diet. MPD^2^ Medium-Production Diet. HPD^3^ High-Production Diet.

**Table 4:** Experiment 1 descriptive statistics per Production Profile (PP) classification and treatment, and *p*-values for the relevant ANOVA analysis.

	Low-Production Diet (LPD)			Medium-Production Diet (MPD)			High-Production Diet (HPD)			ANOVA *p*-Value	
Production Profile classification	3	2	1	3	2	1	3	2	1	Effect of Diet	Effect of PP class
*n*	42	52	40	41	47	38	37	50	39		
Mean entry weight (95% CI) (kg)	**261.4** (245.9–276.8)	**300.1** (288.1–312.2)	**317.1** (305.1–329.2)	**264.3** (249.3–279.3)	**293.0** (280.9–305.1)	**314.0** (299.9–328.1)	**263.6** (247.4–279.7)	**293.0** (280.9–305.1)	**313.2** (300.4–326.0)	0.89	<0.01
Mean carcass entry weight (95% CI) (kg)	143.0 (132.2–153.7)	170.0 (161.5–178.2)	181.6 (173.3–190.0)	145.0 (134.6–155.4)	164.9 (156.5–173.3)	179.5 (169.7–189.3)	144.5 (133.3–155.7)	168.8 (160.8–176.8)	178.9 (170.1–187.8)	0.89	<0.01
Mean carcass weight (95% CI) (kg)	301.1 (286.2–316.0)	338.2 (327.6–348.7)	359.9 (348.9–370.9)	306.5 (293.5–319.5)	342.1 (330.9–353.3)	371.3 (358.5–384.1)	309.9 (295.5–324.2)	348.1 (338.2–358.0)	363.5 (350.6–376.3)	0.25	<0.01
Mean carcass gain (95% CI) (kg)	158.2 (148.6–167.7)	168.3 (162.5–174.1)	178.2 (170.7–185.8)	161.5 (153.3–169.7)	177.2 (171.1–183.2)	191.8 (183.7–200.0)	165.4 (157.1–173.6)	179.3 (172.9–185.7)	184.5 (176.9–192.1)	0.01	<0.01
Mean carcass ADG (95% CI) (kg/d)	1.16 (1.10–1.23)	1.24 (1.19–1.28)	1.30 (1.24–1.35)	1.19 (1.13–1.25)	1.31 (1.27–1.35)	1.39 (1.34–1.45)	1.22 (1.16–1.28)	1.33 (1.28–1.37)	1.35 (1.30–1.40)	<0.01	<0.01

**Table 5:** Mixed-effects regression model of carcass gain (kg) with interaction between Production Profile classification (PP class) and diet in experiment 1.

Predictor	Level	Diet	Coefficient	95% CI ^4^		*p*-Value
Production Profile classification	1	HPD ^1^	4.43	−5.70	14.56	0.39
		MPD ^2^	9.84	−0.45	20.13	0.06
		LPD ^3^	−3.13	−13.27	6.99	0.54
	2	HPD ^1^	1.88	−7.53	11.29	0.70
		MPD ^2^	Reference value	-	-	-
		LPD ^3^	−10.69	−20.04	−1.35	0.03
	3	HPD ^1^	−12.07	−22.37	−1.76	0.02
		MPD ^2^	−14.93	−24.95	−4.92	<0.01
		LPD ^3^	−19.17	−29.15	−9.18	<0.01
Carcass entry weight (kg)			0.04	−0.04	0.11	0.34
DOF ^5^			1.73	1.08	2.39	<0.01
Random effects			Estimate	95% CI		
	Residual		558.16	484.72	642.73	

^1^ High-production diet. ^2^ Medium-production diet. ^3^ Low-production diet. ^4^ Confidence Interval. ^5^ Days on Feed.

**Table 6:** Experiment 2 descriptive statistics per Production Profile (PP) classification and treatment, and *p*-values for the relevant ANOVA analysis.

Diet	Low-Production Diet (LPD)			Medium-Production Diet (MPD)			High-Production Diet (HPD)			ANOVA *p*-Value	
PP class	3	2	1	3	2	1	3	2	1	Effect of Diet	Effect of PP class
*n*	9	17	8	9	16	9	9	18	9		
Mean entry weight (95% CI) (kg)	**199.2** (186.1–212.3)	**212.7** (204.1–221.3)	**224.6** (212.6–236.7)	**200.0** (181.4–218.6)	**211.7** (200.2–223.2)	**229.4** (213.3–245.6)	**205.1** (187.4–222.8)	**215.8** (207.4–224.3)	**225.4** (212.0–239.0)	0.80	<0.01
Mean exit weight (95% CI) (kg)	**380.7** (362.8–398.5)	**440.5** (416.8–464.2)	**469.8** (441.7–497.8)	**415.2** (385.6–444.8)	**457.3** (435.0–479.6)	**481.7** (461.1–502.2)	**413.6** (388.6–438.6)	**467.7** (449.6–485.8)	**502.1** (481.2–523.0)	<0.01	<0.01
Mean live weight gain (95% CI) (kg)	**181.4** (167.3–195.6)	**227.8** (207.7–248.0)	**245.1** (223.6–266.6)	**215.2** (200.4–230.0)	**245.6** (228.5–262.6)	**252.2** (235.3–269.2)	**208.4** (191.2–225.7)	**251.8** (235.8–267.8)	**276.7** (261.6–291.7)	<0.01	<0.01
Mean ADG (95% CI) (kg/day)	**1.28** (1.18–1.38)	**1.60** (1.46–1.75)	**1.73** (1.57–1.88)	**1.52** (1.41–1.62)	**1.73** (1.61–1.85)	**1.78** (1.66–1.90)	**1.47** (1.35–1.59)	**1.77** (1.66–1.89)	**1.95** (1.84–2.05)	<0.01	<0.01
Mean carcass entry weight (95% CI) (kg)	**111.6** (102.5–120.7)	**121.0** (115.0–126.9)	**129.2** (120.9–137.6)	**112.1** (99.2–125.0)	**120.2** (112.3–128.2)	**132.6** (121.4–143.8)	**115.7** (108.4–128.0)	**123.1** (117.2–130.0)	**129.8** (120.4–139.1)	0.80	<0.01
Mean carcass exit weight (95% CI) (kg)	**231.1** (220.7–241.5)	**269.2** (255.1–283.4)	**285.8** (267.6–304.0)	**252.6** (232.7–272.5)	**285.2** (270.0–300.5)	**301.1** (286.8–315.3)	**258.7** (241.2–276.2)	**294.0** (280.0–306.0)	**319.1** (305.1–333.1)	<0.01	<0.01
Mean carcass gain (95% CI) (kg)	**119.5** (111.5–127.5)	**148.3** (136.8–159.8)	**156.6** (143.3–169.8)	**140.5** (130.6–150.3)	**165.0** (153.3–176.7)	**168.5** (156.7–180.3)	**143.0** (129.1–156.9)	**170.8** (160.4–181.3)	**189.3** (179.8–198.8)	<0.01	<0.01
Mean carcass ADG (95% CI) (kg/d)	**0.84** (0.79–0.90)	**1.04** (0.96–1.13)	**1.10** (1.01–1.20)	**1.00** (0.92–1.06)	**1.16** (1.08–1.24)	**1.19** (1.10–1.27)	**1.01** (0.91–1.10)	**1.20** (1.13–1.28)	**1.33** (1.27–1.40)	<0.01	<0.01

**Table 7:** Experiment 2 descriptive statistics per pen by Production Profile (PP) classification and diet, and *p*-values for the relevant ANOVA analysis.

Diet	Low-Production Diet (LPD) Pen			Medium-Production Diet (MPD) Pen			High-Production Diet (HPD) Pen			ANOVA *p*-Value	
Production Profile classification	3	2	1	3	2	1	3	2	1	Effect of Diet	Effect of PP class
*n* (pens)	3	6	3	3	6	3	3	6	3		
Mean daily feed intake (95% CI) (kg)	**8.69** (7.09–10.28)	**10.74** (9.64–11.85)	**11.19** (10.03–12.36)	**9.70** (9.51–9.89)	**10.28** (9.71–10.85)	**11.03** (10.35–11.71)	**8.81** (8.48–9.14)	**10.35** (9.65–11.05)	**10.69** (9.95–11.43)	0.67	<0.01
Mean feed conversion ratio (95% CI) (kg/kg)	**7.04** (6.52–7.56)	**6.70** (6.28–7.13)	**6.27** (6.00–6.55)	**6.44** (6.10–6.79)	**6.04** (5.80–6.28)	**6.17** (5.70–6.64)	**6.41** (5.10–7.71)	**5.83** (5.46–6.20)	**5.47** (5.05–5.90)	<0.01	0.02
Mean carcass feed conversion ratio (95% CI) (kg/kg)	**10.62** (9.69–11.55)	**10.29** (9.67–10.90)	**9.82** (9.75–9.89)	**9.81** (9.20–10.42)	**9.04** (8.63–9.45)	**9.18** (8.36–10.00)	**9.40** (7.34–11.46)	**8.59** (8.17–9.01)	**8.03** (7.53–8.54)	<0.01	0.02

**Table 8:** Mixed-effects regression model of carcass gain (kg) with interaction between Production Profile classification (PP class) and diet in experiment 2.

Predictor	Level	Diet	Coefficient	95% CI		*p*-Value
Production Profile classification	1	HPD ^1^	23.0	7.03	38.96	0.05
		MPD ^2^	1.82	−14.28	17.91	0.83
		LPD ^3^	−9.66	−26.21	6.90	0.25
	2	HPD ^1^	5.46	−7.57	18.49	0.41
		MPD ^2^	Reference value	-	-	-
		LPD ^3^	−16.78	−29.97	−3.60	0.01
	3	HPD ^1^	−21.37	−37.18	−5.55	<0.01
		MPD ^2^	−23.37	−39.28	−7.46	<0.01
		LPD ^3^	−44.27	−60.20	−28.34	<0.01
Carcass entry weight (kg)			0.14	−0.12	0.40	0.30
Random effects			Estimate	95% CI		
	Residual		372.96	284.20	489.44	

^1^ High-production diet. ^2^ Medium-production diet. ^3^ Low-production diet.

**Table 9:** Mixed-effects regression model of carcass feed conversion ratio per pen (kg fed/kg carcass gained) with interaction between Production Profile classification (PP class) and diet in experiment 2.

Predictor	Level	Diet	Coefficient	95% CI		*p*-Value
Production Profile classification	1	HPD ^1^	−0.91	−1.86	−0.04	0.06
		MPD ^2^	0.27	−0.74	1.28	0.60
		LPD ^3^	0.87	−0.08	1.80	0.07
	2	HPD ^1^	−0.42	−1.15	0.30	0.26
		MPD ^2^	Reference value	-	-	-
		LPD ^3^	1.25	0.53	2.00	<0.01
	3	HPD ^1^	0.31	−0.60	1.21	0.50
		MPD ^2^	0.27	−0.74	1.28	0.60
		LPD ^3^	1.50	0.57	2.44	<0.01
Carcass entry weight (kg)			−0.01	−0.05	0.03	0.62
Random effects			Estimate	95% CI		
	Residual		0.40	0.25	0.64	

^1^ High-production diet. ^2^ Medium-production diet. ^3^ Low-production diet.

## Data Availability

The original contributions presented in this study are included in the article. Further inquiries can be directed to the corresponding author.
